# Supplementary material for: CompAIRR: ultra-fast comparison of adaptive immune receptor repertoires by exact and approximate sequence matching
Source: Bioinformatics. 2022 Jul 19;38(17):4230–2. doi: 10.1093/bioinformatics/btac505 (PMC9438946; doi:10.1093/bioinformatics/btac505)
Supplement: btac505_Supplementary_Data [file btac505_supplementary_data.docx]

# **Supplementary Material**


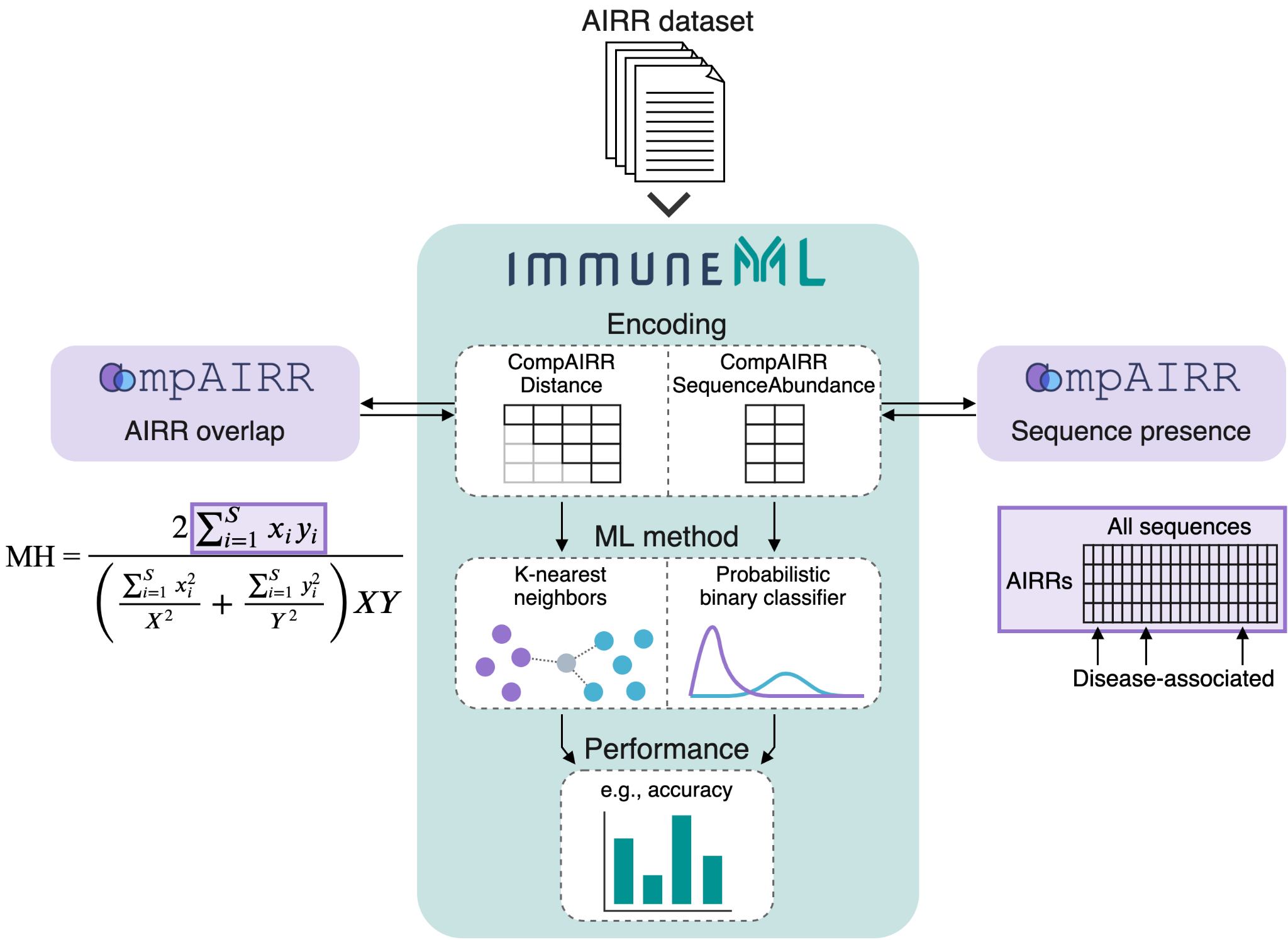


**Supplementary Figure 1 | CompAIRR is integrated with immuneML to speed up core calculations of two different encodings.** Firstly, CompAIRR accelerates the calculation of a Morisita-Horn (MH) distance matrix by calculating the sum of the products of overlapping AIR frequencies between AIRRs (left, highlighted in purple). This distance matrix can subsequently be used in combination with a k-nearest neighbors classifier. Secondly, calculating a sequence presence matrix with CompAIRR (right, highlighted in purple) speeds up an AIRR classification method (Emerson *et al.*, 2017), which was replicated in the immuneML ecosystem (Pavlović *et al.*, 2021) by a sequence abundance encoding (AIRR size and number of disease-associated AIRs per AIRR) and a probabilistic binary classifier.


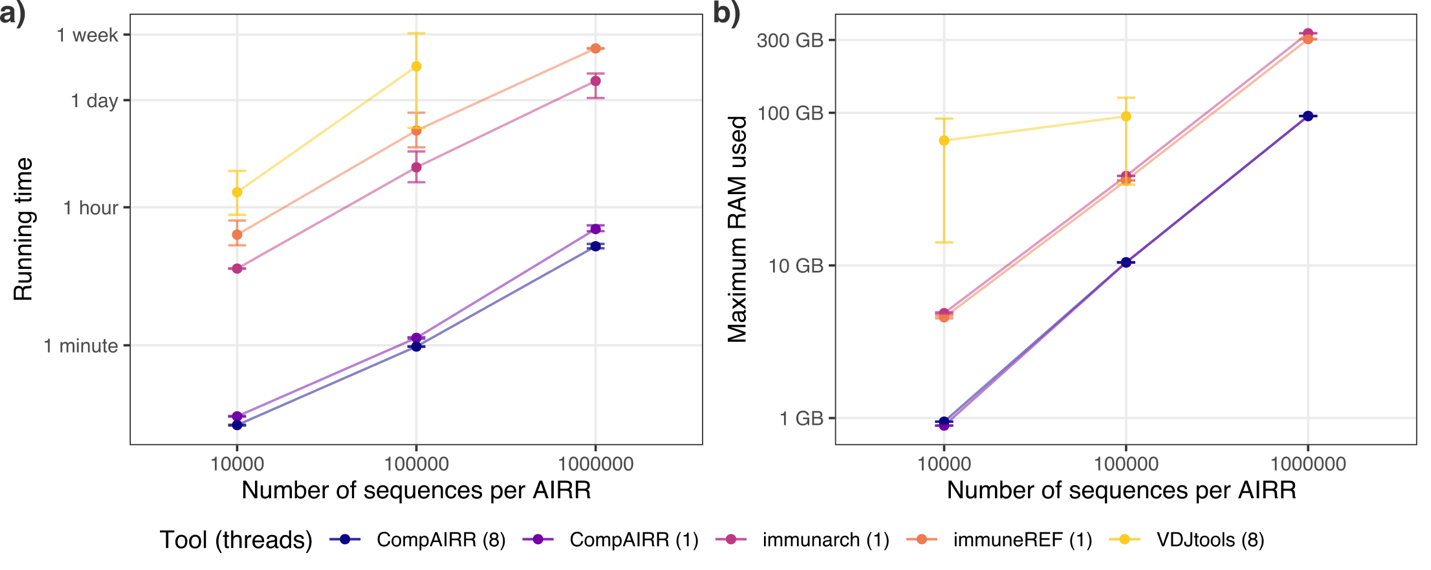


**Supplementary Figure 2 | Benchmarking of (a) running time and (b) maximum RAM usage as a function of the number of sequences per AIRR.** Data shown are mean with error bars showing min/max values across three replicate runs. Datasets consist of 1000 OLGA-generated AIRRs (Sethna *et al.*, 2019) (default human IgH CDR3 model). VDJtools failed to run on the dataset of 10^6^ AIRRs due to memory limitations.


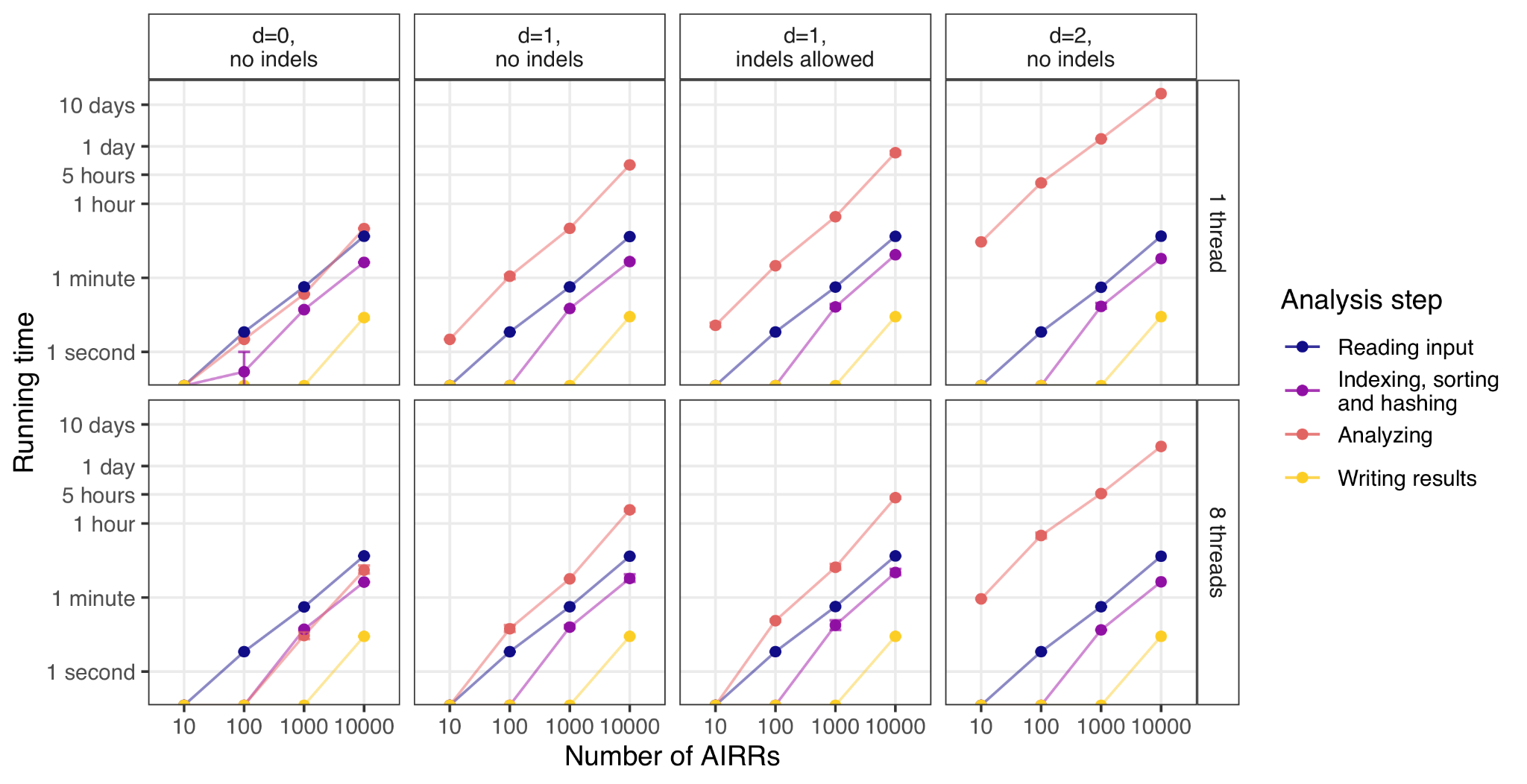


**Supplementary Figure 3 | Running time of CompAIRR analysis steps.** Reading input and analyzing the AIRR overlap are the most computationally intensive steps. When exact sequence matching is used, reading input data takes a relatively large amount of the total running time. As a result, the benefit of multi-threading is more apparent when non-exact matching is used.


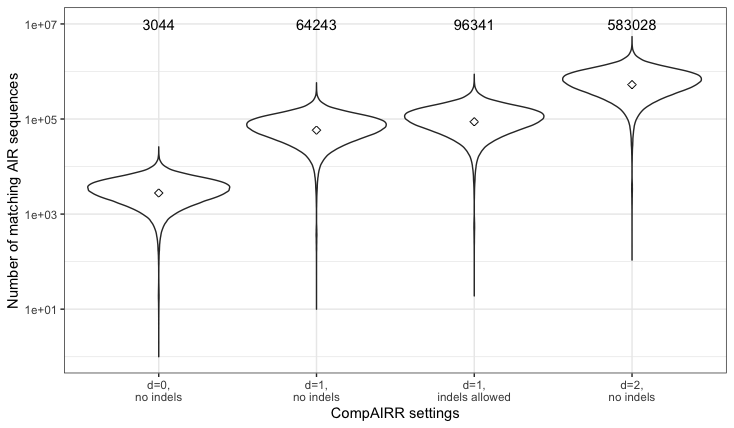


**Supplementary Figure 4 | Number of matches with approximate sequence matching settings.** The number of matching TCRβ AIR sequences between each combination of 710 AIRRs (Emerson *et al.*, 2017) increases when less stringent sequence matching criteria are used. When no or few exact overlapping sequences are found across AIRRs, highly similar sequences may still be present. Medians are represented by rhombuses and their values are displayed above the violin plots.
